# The therapeutic landscape of inherited bleeding disorders in China

**DOI:** 10.1016/j.rpth.2026.103460

**Published:** 2026-03-27

**Authors:** Yun Wang, Feng Xue, Man-Chiu Poon, Renchi Yang

**Affiliations:** 1 State Key Laboratory of Experimental Hematology, National Clinical Research Center for Blood Diseases, Haihe Laboratory of Cell Ecosystem, Tianjin Key Laboratory of Gene Therapy for Blood Diseases, CAMS Key Laboratory of Gene Therapy for Blood Diseases, Institute of Hematology & Blood Diseases Hospital, Chinese Academy of Medical Sciences & Peking Union Medical College, Tianjin, China; 2 Tianjin Institutes of Health Science, Tianjin, China; 3 Departments of Medicine, Pediatrics and Oncology, University of Calgary Cumming School of Medicine, Calgary, Canada

**Keywords:** China, hemophilia, inherited bleeding disorders, tiered healthcare system

## Abstract

The demand for diagnosis and treatment of inherited bleeding disorders, particularly hemophilia, is increasing in China. In recent years, significant progress has been made in the country regarding the development and application of related therapeutics and the construction of the Chinese healthcare delivery system with attention to more efficient, cost-effective and convenient healthcare access to the population in both the remote/rural and urban areas. This article aims to systematically review the current therapeutic landscape of inherited bleeding disorders in China, elucidate the developmental pipeline of investigational domestic novel drugs, and discuss the establishment of a tiered healthcare system for hemophilia in China in line with the developing national healthcare delivery system. Attention to research and development in China have resulted in increased therapeutic product manufacturing capacity, and China is progressively establishing a domestic supply system for therapeutics ranging from basic replacement therapies to advanced treatments including gene therapy for inherited bleeding disorders. Concurrently, the ongoing development of the tiered healthcare system is expected to optimize the allocation of medical resources and enhance the standardization of diagnosis and treatment nationwide. In the future, the successful development of domestic innovative drugs, combined with an efficient and cost-effective healthcare delivery system, will lay a solid foundation for improving treatment outcomes and the quality of life for patients in China.

## Essentials

• The demand for the diagnosis and treatment of inherited bleeding disorders (IBDs), particularly hemophilia, is increasing in China.
• China has made significant progress in developing treatments for IBDs and building its healthcare system.
• China has diverse IBD therapeutics and an evolving tiered hemophilia care system.
• China will continue to refine its IBD diagnosis and treatment system.

## 1 Introduction

Inherited bleeding disorders (IBDs) are a group of diseases caused by genetic defects that lead to dysfunction or deficiency of one or more components in the hemostatic process, thereby increasing the risk of bleeding [1]. These components are involved in multiple steps of hemostasis and primarily include coagulation factor deficiencies, platelet function disorders/inherited thrombocytopenia, and abnormal vascular development. Hemophilia A, hemophilia B, and von Willebrand disease are the most common, constituting over 90% of all IBDs [2].

Globally, the prevalence of hemophilia A is approximately 17.1 cases per 100,000 males and that of hemophilia B is approximately 3.8 cases per 100,000 males [3]. In China, based on the only nationwide survey on the prevalence of hemophilia conducted between 1986 and 1989, the prevalence of hemophilia was 2.73 per 100,000 individuals, with patients with hemophilia A being 80% of the total [4,5]. As the national hemophilia registry system continues to improve, approximately 30,000 to 40,000 patients with hemophilia had been registered in China by 2025, corresponding to an estimated prevalence of 2.1 to 2.9 per 100,000 population. Considering potential underdiagnosis and underreporting, the actual number of patients in China is expected to be higher than the registered figure, given that the globally recognized incidence of hemophilia is approximately 1 in 5000 male births. Nonetheless, although the prevalence rate of IBDs is relatively low, the number of patients in China is substantial due to the large population base of 1.4 billion people. In recent years, there has been increasing attention focused on the care of these patients in China. In 2018, hemophilia was included in the First National List of Rare Diseases issued by National Health Commission. Consequently, the China Hemophilia Treatment Center Capacity Building Project was launched in 2020, aimed to standardize the diagnosis and treatment system for hemophilia.

Traditional management of hemophilia has primarily relied on factor replacement therapy. However, long-term, repeated infusion of coagulation factors not only increases the psychological and economic burden on patients; there is also a risk of inhibitor development [[6], [7], [8]]. The current non-factor and gene therapies have shown promise in reducing bleeding episodes, decreasing the frequency of intravenous infusions and/or providing more convenient administration, thus improving the quality of life and alleviating their psychological burden.

This review aims to elucidate the current status of research and treatment for IBDs in China. The advance in hemophilia therapeutics in China is paralleled by improvement in the hemophilia research and hemophilia care infrastructure in the country over the last several decades, and we are now in the midst of further development of our care system to achieve effective delivery of therapy and care. Attention to research and development has progressively improved the capacity for therapeutic product manufacturing in China. There is increasing availability of domestic plasma-derived and recombinant concentrates as well as more advanced therapeutics including gene therapy to complement the imported products.

## 2 Current Status of Hemophilia Care in China

Since the establishment of the Hemophilia Treatment Center Collaborative Network of China (HTCCNC) in 2004, significant progress has been made in China in the areas of hemophilia diagnosis and treatment networks, registration systems, professional training and networking, guideline development, patient education, and policy support. After the establishment of the HTCCNC, the Hemophilia Training Center program was steadily advanced across the country, ultimately leading to the formation of a nationwide network of hemophilia centers. The current delivery of hemophilia care in China follows a tiered management system, coordinated under the guidance of the National Health Commission and organized through a network of certified treatment centers. The Figure shows the map of China with the distribution of clinics providing various levels of care, including the 6 collaborative World Federation of Hemophilia (WFH) China Hemophilia Training Centers established in 2010 and the WFH International Hemophilia Training Center established in 2025.

**Figure:**
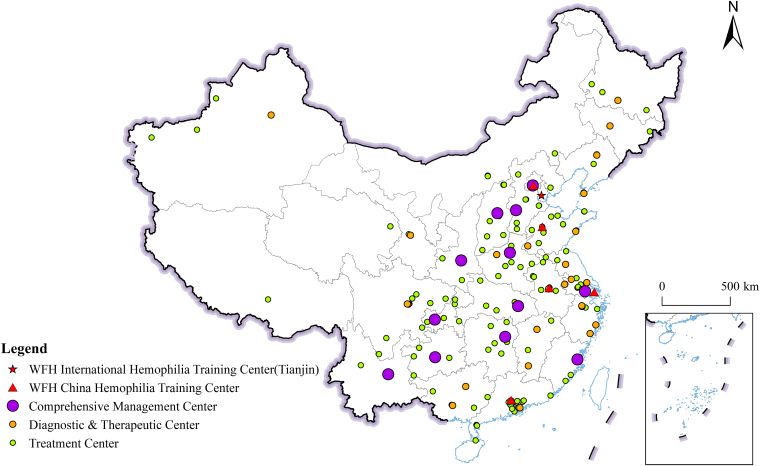
Map of China: distribution of hemophilia treatment and training centers. (A) The 3 levels of hemophilia treatment centers in China: (1) Comprehensive Management Centers: centers with multidisciplinary teams, providing comprehensive care and follow-up; (2) Diagnostic & Therapeutic Centers: centers to provide routine care of inherited bleeding disorders and inhibitor diagnosis, replacement therapy, and management of routine bleedings; (3) Treatment Centers: centers to provide routine and emergency bleeding treatment, and tend to be in rural and less serviced areas with more restricted resources. These 3 levels of centers are interconnected through standardized referral pathways, teleconsultation mechanisms, and data-sharing platforms, enabling both vertical coordination and horizontal collaboration. (B) Tianjin, now designated as a World Federation of Hemophilia (WFH) International Hemophilia Training Center, is 1 of 6 cities (together with Beijing, Jinan, Hefei, Shanghai, and Guangzhou) to establish WFH China Hemophilia Training Centers. All 6 centers are also designated as Comprehensive Management Centers.

A questionnaire-based study revealed that the average annual direct medical cost per patient reaches as high as 450,000 RMB (exchange rate as of February 2026: 1 RMB ≈ $0.14 USD), primarily attributable to expenditures on clotting factor concentrates [9]. Although reimbursement rates vary across different medical insurance schemes, ranging from 50% to 90%, additional financial support mechanisms, including critical illness insurance, medical assistance programs, and charitable aid, are available to help alleviate patient out-of-pocket expenses. The economic burden associated with hemophilia is still substantial. The average annual out-of-pocket medical cost still exceeds 100,000 RMB per patient. Another study focusing on hemophilia A reported that the total annual cost of treatment per patient (including both inpatient and outpatient services), which encompasses factor VIII replacement therapy, was 57,439.4 RMB [7].

In terms of treatment strategy, the 2025 version of the new guidelines shifted the approach to hemophilia care from “on demand treatment” as a passive response to bleeding towards an active strategy of “bleed prevention” and “rebuilding physiological function” [10]. The treatment for most patients with severe hemophilia is gradually transitioning toward a prophylaxis-based model, and the proportion of patients who have used prophylactic treatment is higher in patients with severe and moderately severe hemophilia in all age groups, but particularly in children [11]. Nevertheless, for prophylactic treatment in adults, the use of recombinant factor VIII (rFVIII) or rFIX is not covered by medical insurance. Currently, insurance reimbursement for adult prophylaxis is limited to plasma-derived coagulation factors. The inconvenience associated with the infusion of plasma-derived coagulation factors and financial constraints partially limit the uptake of prophylactic treatment among eligible adult patients, even with help from other financial supporting mechanisms.

Overall, through multidisciplinary collaboration among hematology, pediatrics, laboratory medicine, and surgery departments, as well as patient and national rare diseases organizations, a nationwide, multilevel, and professional hemophilia prevention and treatment system has been preliminarily established. The treatment model has shifted, in addition to change from on-demand therapy to prophylactic treatment, as well as from plasma-derived clotting factors to recombinant clotting factors, and from reliance on imported therapeutic drugs to greater use of domestically developed innovative medications. The following sections will introduce currently available products (Table 1) as well as those under development (Table 2).

**Table 1:** Hemophilia treatment products currently available in China.

	Domestic products	Approved indications in China	Imported products^a^	Approved indications in China
pd-products^b^			NA	NA
	FVIIa	HA/HB patients with inhibitors		
	FVIII	HA		
	FIX	HB		
	PCC^c^	Congenital or acquired deficiencies of FII, FVII, FIX, and FX; coagulation disorders associated with liver disease, anticoagulants and vitamin K deficiency		
rFVIIa				
	Anqixin (Chia Tai Tianqing)	HA/HB patients (aged ≥12 y) with FVIII or FIX inhibitors >5 BU	NovoSeven (Novo Nordisk)	HA/HB patients with FVIII or FIX inhibitors >5 BU; acquired hemophilia; congenital FVII deficiency; Glanzmann's thrombasthenia
SHL-rFVIII				
	Anjiayin (Sinocelltech)	HA patients (pediatric and adult) : on demand treatment, prophylaxis, and perioperative management	Kovaltry (Bayer)	HA patients (pediatric and adult) : on demand treatment, prophylaxis, and perioperative management
	Anhengji (Chia Tai Tianqing)	HA patients (aged ≥12 y): for prophylaxis	Advate (Takeda)	HA patients (pediatric and adult) : on demand treatment, prophylaxis, and perioperative management; HA patients (aged ≥16 y): for prevention of joint damage
	Ronganning (Chengdu Rongsheng)	HA patients (pediatric and adult) : on demand treatment, prophylaxis, and perioperative management	Xyntha (Pfizer)	HA patients (pediatric & adults) : on demand treatment, prophylaxis, and perioperative management
			NovoEight (Novo Nordisk)	HA patients (aged ≥12 y): on demand treatment, prophylaxis, and perioperative management
EHL-rFVIII	NA	NA		
			Esperoct (Novo Nordisk)	HA patients (aged ≥12 y): on demand treatment, prophylaxis, and perioperative management
SHL-rFIX	NA	NA		
			Benefix (Pfizer)	HB patients (pediatric and adult) : on demand treatment, prophylaxis, and perioperative management
EHL-rFIX	NA	NA		
			Alprolix (Sanofi)	HB patients (pediatric and adult) : on demand treatment, prophylaxis, and perioperative management
Non-factor therapies	NA	NA		
			Emicizumab (Roche)	HA patients (pediatric and adult) with/without FVIII inhibitors : prophylaxis
			Marstacimab (Pfizer)	HA/HB patients (aged ≥12 y and weight ≥35 kg) without inhibitors: for prophylaxis
			Fitusiran (Sanofi)	HA/HB patients (aged ≥12 y) with/without inhibitors: prophylaxis
Gene therapy (FIX)			NA	NA
	Xinjiuning (Belief BioMed)	Adults with moderate to severe HB		

BU, Bethesda Unit; EHL, extended half-life; F, factor; HA, hemophilia A; HB hemophilia B; NA, not available; pd, plasma derived; r, recombinant; PCC, prothrombin complex concentrate; SHL, standard half-life.

a The names of the manufacturers are in parentheses.

b There are multiple domestic plasma derived (pd)-FVIII, pd-FIX and pd-PCC products from multiple Chinese manufacturers.

c aPCC (activated prothrombin complex concentrate) is not available in China.

**Table 2:** Hemophilia treatment products currently under development in China.

	Investigational product	Trial registration number^a^	Trial status	Potential indication	Product manufacturer
rFVIII					
	FRSW117	CTR20233517	Phase 3 completed	HA	Gensciences
	FRSW107	CTR20210593	Phase 3 completed	HA	Gensciences
	rhFVIII-Fc	CTR20254859	Phase 3 ongoing	HA	Chengdu Rongsheng Pharmaceuticals Co., Ltd
rFVIIa	rhFVIIa-Fc	CTR20232471	Phase 1b/2 completed	HA/HB patients with FVIII/FIX inhibitors	Gensciences
FVIIIa-mimetic bispecific antibody	SS315	pre-clinical study	NA	NA	NA
FX activator	STSP-0601	CTR20250843	Phase 3 ongoing	HA/HB patients with inhibitors	Staidson (Beijing) BioPharmaceuticals Co., Ltd
Anti-TFPI antibody	KN057	CTR20234254; CTR20234255	Phase 3 ongoing	HA/HB patients with/without inhibitors	Suzhou Alphamab Co., Ltd
	AP017	CTR20231036	Phase 1 ongoing	HA/HB patients with/without inhibitors	Anyuan Pharmaceutical Technology (ShangHai) Co., Ltd
Anti-Protein C Anti-body	SR604	CTR20241608; CTR20254811	Phase 2 ongoing	vWD; HA/HB or congenital FVII deficiency	Shanghai RAAS Blood Products Co., Ltd
Gene therapy					
	GS1191-0445	CTR20250365	Phase 3 ongoing	HA	Grit science, Ltd.
	ZS802	CTR20232175	Phase 1/2 ongoing	HA	Sichuan Real&Best Biotech Co., Ltd
	ZS801	CTR20222717	Phase 1/2 ongoing	HB	Sichuan Real&Best Biotech Co., Ltd
	FT-004	CTR20232648	Active, not recruiting	HB	Frontera Therapeutics (Suzhou) Co., Ltd

CTR, Clinical Trial Registry; F, factor; HA, hemophilia A; HB hemophilia B; NA, not available; rh, recombinant human; TFPI, tissue factor pathway inhibitor; VWD**,** von Willebrand Disease.

a See http://www.chinadrugtrials.org.cn.

## 3 Hemophilia Therapeutic Products Currently Available in China

### 3.1 FVIII/FIX products

Currently, the treatment of hemophilia A and B in China remains predominantly reliant on clotting factor replacement therapy. Both virus-inactivated plasma-derived clotting factors and recombinant clotting factors are used. Numerous domestically manufactured plasma-derived FVIII and FIX clotting factor products are currently marketed in China. Domestic manufacturers include Sinopharm Group (Shanghai), Chengdu Rongsheng, and Sichuan Yuanda Shuyang, among others.

The recombinant clotting factors currently available in China are mostly SHL (standard half-life) and mainly imported but are increasingly complemented with domestically manufactured products. The available imported rFVIII products include octocog alfa (Kovaltry/Bayer and Advate/Takeda), moroctocog alfa (Xyntha, Pfizer), and turoctocog alfa (NovoEight, Novo Nordisk), etc. The first Chinese developed and produced SHL-rFVIII, omfiloctocog alfa (brand name: Anjiayin), manufactured by Beijing SinocellTech Group Co, Ltd, was marketed in 2021. Omfiloctocog alfa is a third-generation B-domain deleted rFVIII. It employs a Chinese hamster ovary cell expression system, without the addition of any human or animal-derived proteins throughout the manufacturing process, effectively reducing immunogenicity and the risk of viral infections. Its baseline half-life determined by chromogenic assay is 11.52 ± 3.61 hours [12]. Phase 3 clinical trials have demonstrated that omfiloctocog alfa exhibits favorable efficacy and tolerability in both prophylaxis and treatment of bleeding episodes in children and adults with hemophilia A. Among 73 previously treated severe hemophilia A patients aged 12 to 65 years without FVIII inhibitor, 78% of patients maintained FVIII levels ≥1% (≥1 IU/dL) 48 hours after the initial dose (49.5 ± 1.10 IU/kg). For patients receiving prophylactic treatment, the mean annualized bleeding rate (ABR) was 2.82 (95% CI, 2.01-3.96). At the same dose range (20-50 IU/kg), the ABR was comparable between the every-other-day regimen and the thrice-weekly regimen. For patients experiencing bleeding episodes, 76.6% of episodes were controlled with a single infusion of omfiloctocog alfa [12]. A study involving 69 previously treated children with severe hemophilia A (6-12 years old) with no history of inhibitor development demonstrated that omfiloctocog alfa also has good efficacy and tolerability, with a FVIII inhibitor incidence of 1.4% [13]. In a 12-month, multicenter real-world study, the median ABR decreased to 0.98 across all age groups, with hemostatic efficacy rated as ‘excellent’ or ‘good’ in 95.6% of bleeding episodes. Over a cumulative observation period exceeding 29,029 exposure days, no inhibitors were detected [14].

In 2023, additional SHL-rFVIII products from Chia Tai Tianqing and Chengdu Rongsheng were successively launched in China. Anhengji (TQG202, generic name: recombinant coagulation FⅧ for injection), developed by Chia Tai Tianqing Pharmaceutical Group, is the first humanized cell line-derived rFVIII marketed in China. Produced by human-derived HEK293 cells as a B-domain deleted rFVIII, Anhengji exhibits a posttranslational modification pattern similar to that of human FVIII, closely resembling endogenous FVIII molecules without carrying nonhuman epitopes, thereby reducing immunogenicity and inhibitor formation. In a prophylaxis study involving 81 patients, the median ABR was 0 (IQR, 0.00-2.14), and the median annualized joint bleeding rate was also 0 (IQR, 0-2.12). The treatment demonstrated a favorable safety profile with no inhibitor development throughout the study [15]. Anhengji also achieved ‘excellent’ or ‘good’ hemostatic efficacy ratings in moderate to severe hemophilia patients in an on-demand treatment study [16]. Anhengji was approved in 2023 for the prevention of bleeding in patients with hemophilia A (congenital FVIII deficiency) aged 12 years and older. In the same year, a recombinant human coagulation FVIII (brand name: Ronganning) independently developed by Chengdu Rongsheng Pharmaceuticals Co, Ltd, was approved for market launch, indicated for the control and prevention of bleeding in patients with hemophilia A (congenital factor VIII deficiency) aged 12 and older [17].

For hemophilia B, plasma-derived FIX concentrates from Shandong Taibang Biological Products Co, Ltd and Sichuan Yuanda Shuyang Pharmaceutical Co, Ltd are used. In addition, there are multiple domestic plasma-derived prothrombin complex concentrates (PCCs) available. Currently, there is no domestically developed rFIX product available in China [18]. For SHL-rFIX, nonacog alfa (Benefix) from Pfizer, approved in China in 2012, is available. On April 23, 2021, the extended half-life (EHL) rFIX eftrenonacog alfa (Alprolix) was approved for marketing in China. It is the world’s first recombinant human FIX-Fc fusion protein.

### 3.2 Recombinant FVIIa

Approximately 20% to 35% of patients with severe hemophilia A and about 10% of those with severe hemophilia B develop inhibitors within 10 to 20 exposure days after initial treatment with factor replacement therapy, with a higher incidence observed in young children with severe hemophilia [19]. For patients with high-titer inhibitors (≥5 Bethesda Unit, BU), bypassing agents such as recombinant activated factor VII (rFⅦa) and activated PCC are recommended [20]. There are currently no approved imported or domestically produced activated PCC products available in China. The imported rFVIIa available in China is NovoSeven (Novo Nordisk). The approved indications include the treatment of bleeding episodes in adults and children with congenital hemophilia and FⅧ or FⅨ high-titer inhibitor, as well as in specific populations with certain rare bleeding disorders, such as acquired hemophilia and congenital FVII deficiency. On the other hand, the first domestically developed rFVIIa (TG203, brand name: Anqixin) was recently approved (July 2025) for the Chinese market [21]. This rFVIIa molecule consists of light and heavy chains comprising a total of 406 amino acids, featuring both *O*-linked and *N*-linked glycosylation, and carries γ-carboxylation modifications at glutamate residues on the N-terminus of the heavy chain. Anqixin exhibits a dual-targeting mechanism of action, promoting thrombin generation through both tissue factor-dependent and tissue factor-independent pathways, facilitating the formation of stable fibrin clots to achieve hemostasis. The phase 3 clinical trial was performed on 60 hemophilia A or B patients with inhibitor titers >5 BU. The hemostatic efficacy rate was excellent at 88.93%, with 78.89% achieving hemostasis within 8 hours, similar to the imported rFVIIa from Novo Nordisk [22]. The overall incidence of treatment-emergent adverse events was 88.33% (53 of 60), and the incidence of serious adverse events (SAEs) was 6.67% (4 of 60), all determined to be unrelated to the study drug [23].

### 3.3 Non-factor therapies

Currently in China, 3 non-factor therapeutic agents are available: the FVIII mimetic emicizumab, the rebalancing agent marstacimab (targeting tissue factor pathway inhibitor) and siRNA drug fitusiran (targeting antithrombin). In China, the approved use of emicizumab is for prophylaxis in children and adults with hemophilia A, with or without FVIII inhibitors. Marstacimab was recently approved in China on November 21, 2025, for prophylactic treatment in patients aged ≥12 years with severe hemophilia A or B without inhibitors [24]. Fitusiran was approved in China on December 15, 2025, for prophylactic treatment of patients with severe hemophilia A or B aged ≥12 years with or without inhibitors. No domestically developed drugs in this class have received approval for clinical use [25].

### 3.4 Gene therapy

In 2019, we initiated Asia’s first clinical study of a liver-targeting adeno-associated virus (AAV) vector-based gene therapy for hemophilia B. This first domestically developed liver-targeting AAV vector-based FIX gene therapy, BBM-H901, dalnacogene ponparvovec, was approved in China in April 2025 for adult patients with severe and moderate hemophilia B [26]. Its phase 1 pilot trial for safety evaluation was conducted on 10 adult patients with hemophilia B in 2019 at the Institute of Hematology & Blood Diseases Hospital, Chinese Academy of Medical Sciences. No infusion reactions occurred in the participants during the infusion process. Two patients experienced grade 2 adverse events related to BBM-H901 (pyrexia and elevated liver enzymes), which resolved after administration of short-term corticosteroids and hepatoprotective agents, respectively, and no inhibitors against the expressed FIX Padua were generated. Vector-derived FIX was detectable as early as 1 day after infusion, with a median time to peak of 5 weeks, achieving a mean FIX:C level of 36.9 IU/dL [27]. A 26-year-old male patient with severe hemophilia B treated with BBM-H901 gene therapy successfully underwent unilateral total knee arthroplasty without receiving exogenous FIX during the perioperative period [28]. The results of the phase 1-3 clinical trials were reported in 2025 [29]. At 52 weeks postinfusion, the mean ABR was reduced to 0.60, with mean coagulation factor levels approaching 40 IU/dL. The primary adverse reaction was manageable transaminitis. Long-term follow-up was conducted on 9 patients from the phase 1 pilot trial with a median follow-up duration of 235 weeks (range, 210-270 weeks). FIX:C levels were maintained at >50 IU/dL in 4 participants and between 10 and 50 IU/dL in 5. None of the 9 patients experienced any bleeding events during this period. One of the 9 participants, whose FIX:C level was 2 IU/dL at 159 weeks posttreatment, subsequently underwent total right knee arthroplasty and initiated prophylactic treatment with rFIX after the surgery [29].

## 4 Therapeutic Products for Hemophilia Currently Under Development in China

### 4.1 EHL-FVIII

Given that the *in vivo* half-life of FVIII is 8 to 12 hours and that of FIX is 18 to 24 hours, patients undergoing clotting factor replacement therapy require frequent infusions. Consequently, research and development into EHL recombinant clotting factors (EHL-rF) has emerged, and there are 2 EHL-rFVIII products currently under development in China.

FRSW117, developed by Gensciences, is a novel long-acting rVIII product that employs a combination of Fc fusion and PEGylation technologies to achieve EHL, enabling once-weekly dosing. Results from the reported phase 1 (NCT04864743) and phase 2 (NCT05265286) clinical trials demonstrate that after single doses of 25 IU/kg and 50 IU/kg, the half-life of FRSW117 was 28.4 hours and 30.8 hours, respectively. This is longer than the 16 to 19 hours observed for other traditional EHL-rFVIIIs [30], though shorter than the 47.0 hours for efanesoctocog alfa (ALTUVIIIO, Sanofi) [31]. Treatment-emergent adverse events occurred in 66.7% of subjects, all of which were grade 1 or 2. No FVIII inhibitors were detected in any subject, and no treatment-emergent anti-PEG-rhFVIII-Fc antibodies or anti-PEG antibodies were observed [32]. In the phase 1 trial, no spontaneous bleeding episodes occurred over 168 hours after a single administration of FRSW117. In the phase 2 trial, 1 spontaneous joint bleed (6.67%) occurred 130.65 hours after the last dose in the 40 IU/kg cohort, along with 1 traumatic oral bleed due to tooth brushing. Both bleeding episodes were resolved with a single dose of FRSW117 (mean dose, 30.01 ± 0.008 IU/kg). A phase 3 study is currently underway in China (NCT06142552). This ‘ultra-long-acting’ rFVIII is expected to reduce dosing frequency, improve treatment adherence, and provide prolonged bleeding protection [33].

Another EHL-rFVIII-Fc fusion protein developed by Gensciences, FRSW107, has completed phase 3 clinical trials. The results from the 119-patient study showed that prophylactic treatment with FRSW107 resulted in a 95.3% reduction in ABR (to mean ABR 1.5 ± 3.8 events), with 63.9% of patients achieving zero bleeds. The mean annualized joint bleeding rate was similarly reduced by 95.8% from baseline. Treatment-related adverse events occurred in 19 patients and were all tolerable. The results demonstrate that FRSW107 is well-tolerated and highly effective for both prophylactic and on-demand treatment of bleeding events in previously treated adolescents and adults with severe hemophilia A [34].

### 4.2 FVIIIa-mimetic bispecific antibody

SS315 is a novel symmetric FVIIIa-mimetic bispecific antibody that links FX and FIX to promote coagulation for the treatment of hemophilia A. In humanized hemophilia A mouse models, doses of 0.5 to 1.0 mg/kg achieved hemostatic effects comparable to 3.0 mg/kg emicizumab. Results from acquired hemophilia A cynomolgus monkey models also support the superior *in vivo* potency of SS315. Low doses of SS315 (0.5 and 2.0 mg/kg) shortened activated partial thromboplastin time and reduced blood loss and bleeding time. However, no clinical trial information has been released to date [35].

### 4.3 FX activator

To address acute bleeding in patients with hemophilia A or B with inhibitors, FX activators have been developed. The domestically developed specific FX activator, STSP-0601, is a heterotrimeric glycoprotein derived from the venom of *Daboia russelii siamensis*, a subspecies of Russell’s viper. It is isolated and purified through methods including ion-exchange chromatography, mixed-mode chromatography, and gel filtration. STSP-0601 promotes rapid hemostasis by specifically activating coagulation FX. Phase 1 clinical trials indicate that STSP-0601 specifically activates FX in a dose-dependent manner, with no allergic reactions or neutralizing antibodies detected. No thrombotic events occurred [36], further demonstrating its favorable safety profile. The phase 2 clinical trial showed that the effective hemostasis rate was 94.07% (95% CI, 88.66-97.41%) in the 0.10 U/kg dose group and 96.48% (95% CI, 92.89-98.57%) in the 0.16 U/kg dose group. No SAEs or thromboembolic events related to the investigational drug were reported, and no efficacy-impairing anti-drug antibodies were observed [37]. Future studies with larger sample sizes and longer follow-up periods are needed to further investigate its efficacy and safety, providing a hemostatic option for patients with hemophilia and inhibitors [38].

### 4.4 Anti-tissue factor pathway inhibitor antibody

KN057, independently developed by Suzhou Alphamab Co, Ltd, is a monoclonal antibody targeting tissue factor pathway inhibitor (TFPI) designed for prophylactic treatment in patients with hemophilia A or B, with or without inhibitors. It represents China’s first monoclonal antibody directed against TFPI. By specifically binding to and neutralizing TFPI, KN057 alleviates its inhibitory effects on FXa and the tissue factor/FVIIa complex, thereby sustaining thrombin levels and achieving prophylactic hemostasis. *In vitro* experiments confirmed that KN057 promotes thrombin generation in plasma samples from patients with hemophilia. In a phase 1 clinical trial involving healthy adult male participants, single subcutaneous administration of KN057 (0.05-2.5 mg/kg) showed a favorable safety profile. At doses of 1.0 and 2.5 mg/kg, KN057 had a half-life of approximately 1 week. KN057 supports a once-weekly subcutaneous dosing regimen [39]. In a phase 2 clinical trial on 24 patients with hemophilia A or B with treated bleeds, with or without inhibitors, no thrombotic events or KN057-related SAEs occurred in any of the participants. The reduction in ABR compared to baseline ranged from 30.4% to 92.1% across the groups.. During a 24-week treatment period, in the 1.5 mg/kg dose group, the median ABR was 0.0 (range, 0-2.1) in hemophilia patients with inhibitors and 1.1 (range, 0.0-18.0) in those without inhibitors. Thus, KN057 has a favorable safety profile and can effectively reduce ABR regardless of the patient’s inhibitor status. Currently, KN057 has progressed to phase 3 clinical studies (CTR20234254; CTR20234255) [40].

### 4.5 Anti-protein C antibody

SR604 is a humanized chimeric antibody developed by Shanghai RAAS Blood Products Co, Ltd that selectively blocks the anticoagulant activity of human activated protein C (APC). Designed based on the murine monoclonal antibody HAPC1573, SR604 exhibits approximately 60-fold greater affinity for human APC compared with HAPC1573. In both *in vitro* and *in vivo* studies, SR604 dose-dependently shortened the clotting time in plasma deficient of various coagulation factors by blocking the anticoagulation activities of APC. In humanized hemophilic mouse models, SR604 reduced tail bleeding time in hemophilia A or B mice, with efficacy comparable to intravenous administration of plasma-derived FVIII or prothrombin complex concentrate. Additionally, it mitigated joint bleeding and pathology in a knee injury hemophilic mice model. Similar to HAPC1573, SR604 did not impair the cytoprotective and endothelial barrier functions of APC. In the cynomolgus monkeys, at a dose of 0.3 mg/kg, the half-life of SR604 was 171 hours when administered intravenously and 202 hours when administered subcutaneously. The bioavailability after subcutaneous injection was 106% [41].

On November 6, 2024, during the 8th China International Import Expo, Dr Jun Xu presented the phase 1 clinical trial results of SR604 (unpublished data) showing favorable safety and tolerance. SR604 therefore holds promise as a novel targeted therapy for the prophylaxis and treatment of hemophilia A or B. Phase 2 clinical trials are currently underway (CTR20241608; CTR20254811) for the following indications: prophylaxis of bleeding episodes in patients with von Willebrand disease and prophylaxis of bleeding episodes in patients with hemophilia A or B, as well as those with congenital FVII deficiency [42].

### 4.6 Gene therapy

Two hemophilia A and 2 hemophilia B gene therapies are currently under development in China, as shown in Table 2. An additional gene therapy for hemophilia B, FT-004, is not described in detail in this section because the trial is not yet recruiting subjects.

GS1191-0445 (formerly known as GS001) is an investigational AAV-based gene therapy drug independently developed by Grit Science, Ltd‌ for hemophilia A. In the medium-dose cohort (3×10^12^ vector genomes [vg]/kg), after up to 52 weeks of follow-up, adult Chinese patients with severe or moderate to severe hemophilia A achieved a FVIII activity level of 77.0 IU/dL (range, 5.9-324.2 IU/dL), along with a 99.1% reduction in ABR. For the 6 severe hemophilia A patients receiving the low-dose regimen (2×10^12^ vg/kg), the median follow-up period was 156 weeks (range, 144-208 weeks), and the median FVIII activity was 5.6 IU/dL (range, 0.5-52.0) at week 144. For the 6 severe hemophilia A patients receiving the high-dose regimen (4×10^12^ vg/kg), the median follow-up period was 110.5 weeks (range, 104-130 weeks), and the median FVIII activity was 42.7 IU/dL (range, 29.4-92.1) at week 104. The overall safety profile was favorable. In the low-dose cohort, the most common treatment-related adverse events were elevations in alanine aminotransferase and lactate dehydrogenase, each occurring in 3 of 6 (50%) patients, all of which were grade 1-2 in severity. In the high-dose cohort, the most common adverse event was an elevated FVIII:C level (>150 IU/dL), observed in 6 of 6 (100%) patients, predominantly occurring within the first 3 weeks postinfusion. This was followed by alanine aminotransferase elevations in 5 of 6 (83.3%) patients and aspartate aminotransferase elevations in 3 of 6 (50%) patients; the majority of these events were grade 1. One patient in the low-dose cohort experienced SAEs of elevated aspartate aminotransferase (grade 1) at week 16, which resolved after treatment with methylprednisolone. In the high-dose cohort, 2 patients experienced SAEs of elevated FVIII:C (grade 1) on days 14 and 21 postinfusion [43]. Enrollment of a total of 62 subjects across 3 studies has been completed, with the longest treatment duration exceeding 221 weeks. A reduction in ABR of approximately 99% was observed (unpublished data).

ZS802 is an investigational recombinant AAV gene therapy for hemophilia A, originally developed by Sichuan Real&Best Biotech Co, Ltd. ZS802 utilizes the world’s smallest liver-specific promoter, which overcomes the packaging challenge of viral vectors for large genes. It incorporates an optimized FVIII gene sequence and a viral serotype preferentially selected based on the characteristics of the Chinese population, aiming to cover a broader patient base. It is currently undergoing phase 1/2 clinical trials (CTR20232175).

ZS801 is an AAV vector-mediated gene therapy drug independently developed by Sichuan Real&Best Biotech Co, Ltd for hemophilia B. Early results from an investigator-initiated trial showed that a patient, who was originally ineligible for conventional gene therapy due to the presence of neutralizing antibodies against another recombinant AAV serotypes, achieved an increase in clotting FIX activity level from <1 IU/dL to the range of 5 to 40 IU/dL after 7 weeks of low-dose (2.0×10^12^ vg/kg) ZS801 treatment. A clinical trial of the drug is currently recruiting participants (CTR20222717).

## 5 Hierarchical Diagnosis and Treatment System for Hemophilia in China

Over the past decade, significant progress has been made in China’s hemophilia diagnosis and treatment system. However, challenges remain in the accessibility of treatment, the availability of clotting factors, and regional disparities in reimbursement rates [44]. To enhance healthcare security for hemophilia patients, the Chinese government has actively advanced the development of the hemophilia diagnosis and treatment system through the establishment of a hierarchical diagnosis and treatment framework for hemophilia. Drawing on the European standards for hemophilia center development and the Asia-Pacific guidelines for hemophilia prevention and treatment, and in light of China’s socioeconomic and cultural conditions [45,46], HTCCNC formulated the Standards for the Establishment of Hemophilia Centers in China in 2019. After addressing issues identified during the evaluation process and referencing the 2023 Canadian and European standards for hemophilia center development [47,48], the 2024 Edition of the Standards for the Establishment of Hemophilia Centers was established [49].

To achieve the preventive and therapeutic goal of “localized management and standardized diagnosis and treatment,” HTCCNC and the China Alliance for Rare Diseases jointly launched the “China Hemophilia Center Construction Project” in 2020. We classify hemophilia centers into 3 tiers: Comprehensive Management Centers, Diagnostic & Therapeutic Centers, and Treatment Centers. Comprehensive Management Centers provide holistic, multidisciplinary diagnosis, treatment, and long-term care for hemophilia patients. Diagnostic & Therapeutic Centers are responsible for the diagnosis and essential multidisciplinary treatment of hemophilia, while Treatment Centers focus on delivering fundamental care and emergency interventions. The different functions of the tertiary centers are briefly summarized in the Figure [50]. This tiered system for hemophilia center development not only enhances healthcare accessibility in remote and rural areas but also supports continuity of care throughout the patient journey. Rather than operating in isolation, the 3 levels are interconnected through standardized referral pathways, teleconsultation mechanisms, and data-sharing platforms, enabling both vertical coordination and horizontal collaboration. This integrated network approach ensures the delivery of efficient and high-quality medical services. At the end of 2025, a total of 192 hospitals had undergone on-site evaluations and passed accreditation, including 12 comprehensive care centers, 39 diagnosis and treatment centers, and 141 treatment centers (see the Figure for the distribution of these accredited centers). The development of China’s hemophilia diagnosis and treatment system has benefited from assistance from many countries as well as the WFH and a number of international foundations and has achieved remarkable progress over recent decades. The establishment of hemophilia centers in China, along with the resulting scientific research and clinical achievements, ultimately benefits hemophilia patients worldwide.

## 6 Conclusion

Over the past few decades, remarkable progress has been made in the treatment of IBDs in China. In the field of hemophilia, for example, not only have traditional replacement therapies such as clotting factor concentrates become stably supplied and widely accessible, but the advent of gene therapy has also ushered in a new era. Parallel to these therapeutic innovations, a hierarchical diagnosis and treatment system with Chinese characteristics has been established and refined. The implementation of this system will significantly improve the accessibility of diagnosis, standardization of treatment, and continuity of comprehensive management for patients with hemophilia, especially those in remote and rural areas. Another milestone achieved is the recent designation, on December 3, 2025, of the Thrombosis and Hemostasis Diagnosis and Treatment Center/Tianjin Hemophilia Center of the Institute of Hematology & Blood Diseases Hospital, Chinese Academy of Medical Sciences, as the WFH International Hemophilia Training Centre (WFH-IHTC) (see Figure), becoming a component of the IHTC Fellowship Program. In the future, China needs to accelerate pioneering innovations and promote independent research and development of cutting-edge therapies, including gene editing. We also need to further refine the hierarchical diagnosis and treatment strategy for hemophilia and build a new comprehensive prevention and treatment framework for IBDs.
